# A Systematic Review of Recruitment and Retention Within Randomised Controlled Trials of Adults With Down Syndrome

**DOI:** 10.1192/bjo.2024.271

**Published:** 2024-08-01

**Authors:** Aimee Wilkinson, Alba Realpe, Dheeraj Rai

**Affiliations:** 1 Centre for Academic Mental Health, University of Bristol, Bristol, United Kingdom

## Abstract

**Aims:**

Adults with Down syndrome (DS) face significant health inequalities and are at increased risk of numerous health concerns. Despite the need, there is a lack of high-quality randomised trial evidence and clinical interventions for people with DS are largely based on consensus guidelines or clinician preferences. As life-expectancy of those with DS increases, the research gap continues to widen.

There is a perception that randomised controlled trials (RCTs) involving people with DS may be hard to carry out due to difficulties in recruitment and retention of participants. However, there is no scientific literature exploring this topic. This systematic review aimed to assess planned vs actual recruitment and retention in RCTs involving adults with DS, and to summarise reported facilitators and barriers to participation of adults with DS in relevant trials.

**Methods:**

The MEDLINE, PsycINFO, EMBASE databases were searched systematically to retrieve all RCTs involving adults with DS aged 16 years or older published from 01.11.1961 to 15.12.2023. Ongoing RCTs were identified from trial registries and searches were supplemented by review of reference lists. Data extraction is ongoing but seeks to elicit details of trial design; planned and achieved recruitment sample size; planned and achieved retention rate, and any specific recruitment or retention strategies described. Risk of bias analysis was not relevant to the research question and so not performed. The review was prospectively registered on Prospero (CRD42023447126).

**Results:**

The database searches retrieved 1,825 results. Post deduplication, 1,518 articles underwent title and abstract screening, of which 82 full texts were reviewed. 53 papers were included in the final analysis, reflecting 47 RCTs involving 1,772 individuals. Commonly studied interventions included exercise programmes for physical fitness and pharmaceuticals that may augment neuropsychological function. Studies typically reported small sample sizes at the point of randomisation (mean = 38.5, SD = 49.6), with over half reporting a sample size of n < 50. A significant number of studies reported difficulty recruiting and retaining participants (detailed data will be available in the poster). Of the minority of articles that reported power calculations, several reported failure to meet target sample size.

**Conclusion:**

Initial results point to a paucity of high-quality, large-scale RCTs involving adults with DS and challenges related to recruitment of participants. The results may aid development of strategies that allow clinical trial teams to overcome challenges in recruitment and retention in RCTs, and may eventually contribute to the improved health and wellbeing of adults with DS.

